# Changes in serum vitamin D and PTH values using denosumab with or without bisphosphonate pre-treatment in osteoporotic patients: a short-term study

**DOI:** 10.1186/s12902-015-0077-3

**Published:** 2015-12-15

**Authors:** Yukio Nakamura, Mikio Kamimura, Shota Ikegami, Keijiro Mukaiyama, Shigeharu Uchiyama, Akira Taguchi, Hiroyuki Kato

**Affiliations:** Department of Orthopedic Surgery, Shinshu University, Asahi 3-1-1, Matsumoto, 390-8621 Japan; Center of Osteoporosis and Spinal Disorders, Kamimura Orthopaedic Clinic, Kotobuki 595-17, Matsumoto, 399-0021 Japan; Department of Oral and Maxillofacial Radiology, Matsumoto Dental University, Gobara 1780, Shiojiri, 399-0781 Japan

**Keywords:** Osteoporosis, Denosumab, Bisphosphonate, Bone turnover markers

## Abstract

**Background:**

Denosumab is a fully human monoclonal antibody that inhibits receptor activator of nuclear factor kappa-β ligand (RANKL). Previous reports have shown that denosumab treatment of osteoporotic patients decreases bone resorption and fracture risk, but there have been no clinical studies on changes in bone turnover markers, 1,25(OH)_2_D_3_, or parathyroid hormone (PTH) in denosumab therapy with or without bisphosphonate (BP) pre-treatment in Japan.

**Methods:**

Here, we report such findings in 22 patients (11 in the denosumab alone group and 11 in the BP pre-treated group) with osteoporosis following 4 months of treatment. Bone metabolism had been inhibited by prior BP administration in the BP pre-treated group.

**Results:**

The bone resorption markers serum tartrate-resistant acid phosphatase type 5b and urinary type I collagen cross-linked N-telopeptide were significantly decreased from baseline values for the entire study period in both groups. The bone formation marker bone alkaline phosphatase was significantly decreased at 4 months in the denosumab alone group only, and N-terminal propeptide of type 1 procollagen was significantly decreased at 2 and 4 months in the denosumab alone group versus no remarkable change in the BP pre-treated group. In the denosumab alone group, 1,25(OH)_2_D_3_ and PTH were significantly increased at 1 week and decreased gradually thereafter, but these did not change notably in the BP pre-treated group.

**Conclusions:**

Our results suggest that treatment with denosumab causes a strong inhibitory effect on bone resorption markers and mild inhibitory effect on bone formation markers. 1,25(OH)_2_D_3_ and PTH were significantly increased by denosumab but these did not change in the BP pre-treated group.

**Trial registration:**

Current Controlled Trials NCT02156960. Registered 31 May 2014.

## Background

Osteoporosis is a major health concern, especially in elderly women, that carries an increased incidence of bone fracture and ensuing morbidity. Thus, the prevention of fractures is the primary therapeutic goal for this condition [1].

Recent treatments for osteoporosis have been based on our current understanding of bone biology. Receptor activator of nuclear factor-kB ligand (RANKL) is a cytokine that is essential for osteoclast differentiation, activation, and survival [2]. Denosumab, a fully human monoclonal antibody against RANKL shown to selectively inhibit osteoclastogenesis, was approved for use in Japan in 2013. As severe osteoporosis in RANKL transgenic mice was reversed by denosumab administration [3], RANKL appears to be an ideal target for osteoporosis treatment.

In one report, denosumab therapy resulted in a significant, early, and sustained increase in bone mineral density (BMD) and enhanced bone strength in the improvement of both cortical and trabecular bones [4]. Another review described that denosumab treatment for 6 years maintained a low fracture incidence, reduced bone turnover, and an increase in BMD [5]. There have been some reports comparing bisphosphonate (BP) and denosumab therapy after BP pre-treatment in osteoporosis [6–8], wherein denosumab increased BMD and inhibited bone resorptive markers more than BP treatment. In patients previously treated with BP, denosumab treatment resulted in higher BMD increases and greater decreases in bone resorption markers than BP alone [6–9].

The major form of vitamin D in the serum is 25(OH)D, which is its principal storage conformation whose concentration is approximately 1000 times higher than that of 1,25(OH)_2_D_3._ The status of vitamin D in the body is typically evaluated using the stored form of serum 25(OH)D [10]. The 1,25(OH)_2_D_3_ form of vitamin D exerts various major effects on vitamin D metabolism. 1,25[OH]_2_D_3_ is tightly regulated by parathyroid hormone (PTH), fibroblast growth factor (FGF)-23, and other hormones, as well as by cytokines [11]. As it is generally considered that serum 1,25[OH]_2_D_3_ level is not altered in normal conditions, there have been no reports on the regulation of serum 1,25[OH]_2_D_3_ and PTH during osteoporosis treatment with denosumab.

While both are anti-resorptive drugs, BP and denosumab have different action mechanisms in osteoporosis. BP functions after bone deposition and has a long half-life and duration of anti-absorptive effects. Therefore, bone metabolism may be affected by BP pre-treatment, although there have been no reports on whether prior treatment with BP affects serum PTH or vitamin D levels during treatment with denosumab.

In this study, we examined the clinical results of 4 months of denosumab treatment with or without BP pre-treatment on bone turnover markers, serum Ca, 1,25(OH)_2_D_3_, and PTH in Japanese osteoporotic patients.

## Methods

Twenty-two patients with osteoporosis (16 women and 6 men) were recruited for this study at our institutions between July 2013 and April 2014. The cohort’s average age was 74.6 years. The patients were divided into 2 groups of 11 patients each for the denosumab alone (7 women and 4 men; mean ± standard deviation (SD) age: 76.3 ± 7.0 years in women and 69.8 ± 9.2 years in men) and BP pre-treated (2 men and 9 women; mean ± SD age: 75.9 ± 3.4 years in women and 69.0 ± 5.0 years in men) groups. All patients were diagnosed as having primary osteoporosis. Patients in the denosumab alone group had no history of medication that may have affected bone or calcium (Ca) metabolism, while those in the BP pre-treated group had been taking oral BP for at least 6 months prior to this study. The diagnosis of primary osteoporosis was made in accordance with the revised criteria established by the Japanese Society of Bone and Mineral Research [12]. We gave daily Ca and vitamin D supplements to all patients during the denosumab administration period.

Serum Ca was corrected with serum albumin (the reference range 8.5–10.2 mg/dL). Serum bone alkaline phosphatase (BAP) (the reference range in postmenopausal women 3.8–22.6 μg/L) and N-terminal propeptide of type 1 procollagen (P1NP) (the reference range in postmenopausal women 27.0–109.3 ng/mL) were measured as bone formation markers using a chemiluminescent enzyme immunoassay and an antibody radioimmunoassay, respectively. Serum tartrate-resistant acid phosphatase (TRACP)-5b (the reference range in women 120–420 mU/dL) and urine N-terminal telopeptide of type I collagen (NTX) (the reference range in postmenopausal women 14.3–89.0 nmolBCE/mmol・CRE) (Osteomark, Osteox International, Seattle, WA) were measured as markers of bone resorption using the enzyme-linked immunosorbent assay (ELISA). Serum whole PTH (9–39 pg/mL) and 1,25(OH)_2_D_3_ (20.0–60.0 pg/mL) were measured by immunoradiometric assays. Each marker was measured just prior to denosumab administration and at 1 week, 1, 2, and 4 months of denosumab treatment. After overnight fasting, serum and first void urine samples were collected between 8:30 a.m. and 10:00 a.m. Immunoassays were performed by SRL, Inc. (Tokyo, Japan).

Bone mineral density (BMD) was measured using a Dual-energy X-ray Absorption (DXA) fan-beam bone densitometer (Lunar Prodigy; GE Healthcare Bio-Sciences Corp., Piscataway, NJ, USA) at the L1-4 levels of the posteroanterior spine and bilateral hips.

In both groups, we compared the changes in each marker at each time point (at first administration of denosumab and at 1 week, 1, 2, and 4 months afterwards) using linear mixed models and Holm’s correction method for multiple comparisons. Each marker value was individually adopted as a response variable: the timing of the measurement was used as a fixed effect, while the individuality of the measurement was adopted as a random effect. Comparisons between the markers of both groups at each measuring point were performed using Welch’s *t*-test. *P*-values of < 0.05 were considered to be statistically significant. Statistical analyses were performed using the statistical package R, version 3.0.1 (R Development Core Team, http://www.r-project.org).

The authors received no hospitality, honoraria, or travel expenses via the companies that market denosumab. This study was approved by the institutional ethical review board at Shinshu University School of Medicine and Show Inan General Hospital prior to its start and written informed consent was obtained from all subjects.

## Results

### Serum corrected calcium level

**Fig. 1:**
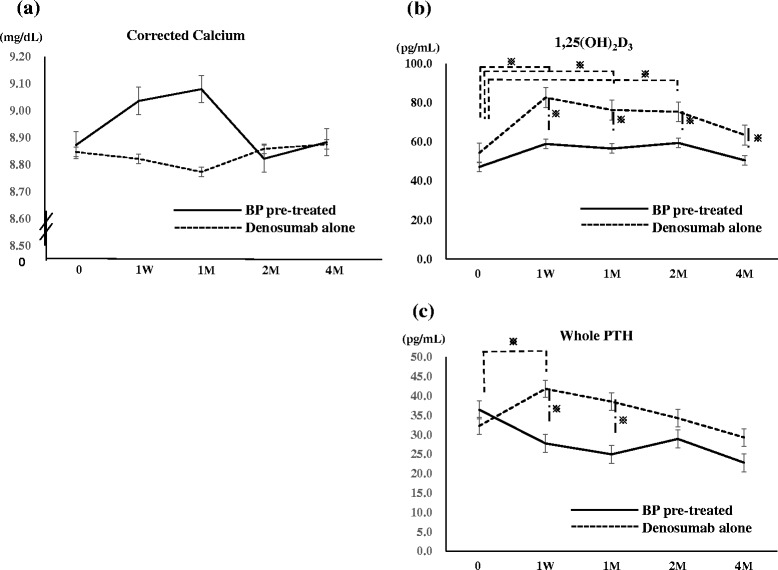
Low Ca stimulates PTH and 1,25(OH)_2_D_3_ expression. **a** Serum Ca in the denosumab alone group did not show any significant changes, but was decreased at 1 week and 1 month followed by a return to baseline levels at 4 months. In the BP pre-treated group, Ca was increased at 1 week and 1 month and gradually decreased thereafter. Group comparisons showed no significant differences at any time point. Straight line: Pre-treated BP group, dotted line: Denosumab alone group. **b** In the denosumab alone group, serum 1,25(OH)_2_D_3_ was significantly increased from 1 week to 2 months, and then gradually decreased thereafter. In the BP pre-treated group, serum 1,25(OH)_2_D_3_ did not significantly change during the study period. Group comparisons showed significant differences from 1 week to 4 months. Straight line: Pre-treated BP group, dotted line: Denosumab alone group. Asterisks indicate significant differences. **c** In the denosumab alone group, whole PTH was significantly increased at 1 week, and then gradually decreased thereafter. In the BP pre-treated group, PTH did not significantly change during the study period. Group comparisons showed significant differences at 1 week and 1 month. Straight line: Pre-treated BP group, dotted line: Denosumab alone group. Asterisks indicate significant differences

Serum Ca level did not change significantly in either group. The changes in adjusted Ca were within the reference range. However, whereas Ca was decreased at 1 week and 1 month before returning to baseline levels at 2 months in the denosumab alone group, it was increased in the BP pre-treatment group at 1 week and 1 month (Fig. 1a).

### Bone turnover markers

#### Bone resorption markers

**Fig. 2:**
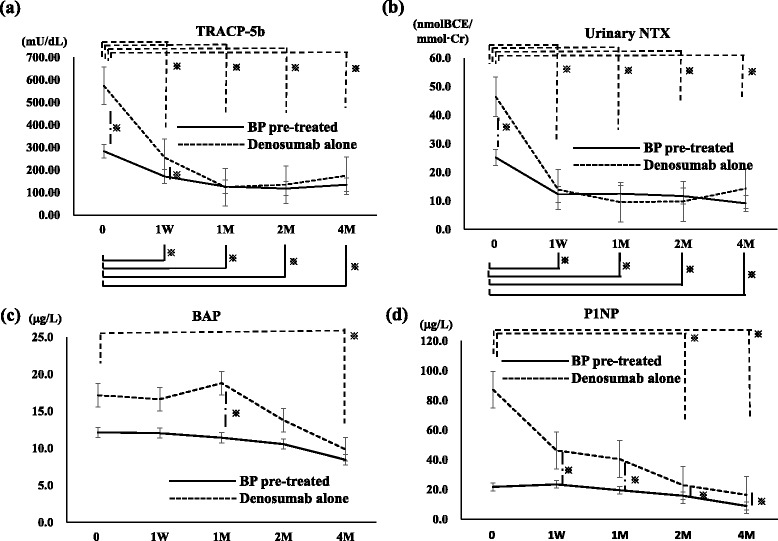
Bone absorption markers were significantly inhibited in the early stages of denosumab administration, whereas bone formation markers were gradually inhibited by denosumab. **a** In the denosumab alone group, serum TRACP-5b was significantly inhibited from 1 week to 4 months. TRACP-5b reached its minimum value at 1 month. In the BP pre-treated group, TRACP-5b was also significantly inhibited from 1 week to 4 months, albeit less than in the denosumab alone group. Group comparisons showed significant differences before administration and at 1 week of administration. Straight line: Pre-treated BP group, dotted line: Denosumab alone group. Asterisks indicate significant differences. **b** In the denosumab alone group, urinary NTX was significantly inhibited from 1 week to 4 months. Urinary NTX reached its minimum value at 2 months. In the BP pre-treated group, urinary NTX was also significantly inhibited from 1 week to 4 months, albeit less than in the denosumab alone group. Group comparisons showed a significant difference before administration. Straight line: Pre-treated BP group, dotted line: Denosumab alone group. Asterisks indicate significant differences. **c** In the denosumab alone group, BAP peaked at 1 month, but then decreased to a significant value at 4 months. In the BP pre-treated group, BAP did not change significantly during the observation period. Group comparisons showed a significant difference at 1 month. Straight line: Pre-treated BP group, dotted line: Denosumab alone group. Asterisks indicate significant differences. **d** In the denosumab alone group, P1NP was significantly decreased at 2 and 4 months. In the BP pre-treated group, P1NP did not change significantly during the observation period. Group comparisons showed significant differences from 1 week to 4 months. Straight line: Pre-treated BP group, dotted line: Denosumab alone group. Asterisks indicate significant differences

Serum TRACP-5b and urinary NTX were significantly lower in the BP pre-treated group before denosumab administration. However, the resorption markers were nonetheless significantly inhibited by denosumab at each time point in both groups (Fig. 2a and b). In the groups, serum TRACP-5b and urinary NTX were significantly inhibited from as early as 1 week to 4 months. These results indicated an immediate and strong anti-resorptive effect of denosumab, even in the BP pre-treated group (Fig. 2a and b).

#### Bone formation markers

Similarly to bone resorption markers, in the BP pre-treated group, BAP and P1NP were at notably lower values compared with the denosumab alone group at treatment onset. In the denosumab alone group, BAP rose slightly at 1 month but then gradually decreased until 4 months after initiation of treatment to a significant degree. P1NP was markedly decreased at 1 week and became significantly lower at 2 and 4 months (Fig. 2c). In the BP pre-treatment group, these bone formation markers had decreased slightly by the end of the observation period (Fig. 2c and d). The values of BAP at 1 month and P1NP at 1 week to 4 months were significantly higher in the denosumab alone group (Fig. 2c and d). These results indicated that following pre-BP treatment, the inhibitory effects on bone formation markers by denosumab were much less pronounced than those in the denosumab alone group.

### Serum 1,25(OH)_2_D_3_ and whole parathyroid hormone (PTH)

In the denosumab alone group, serum 1,25(OH)_2_D_3_ was significantly increased from as early as 1 week to 2 months, peaked at 1 week after denosumab administration, and then gradually decreased. Similarly, whole PTH was significantly increased at 1 week and slowly decreased thereafter (Fig. 1b and c).

In the BP pre-treated group, serum 1,25(OH)_2_D_3_ level was increased slightly up to 4 months. Whole PTH level did not increase, and in fact decreased slightly, but not significantly, during the observation period (Fig. 1b and c).

Before denosumab administration, there were no significant differences in baseline levels of 1,25(OH)_2_D_3_ or whole PTH between groups. However, values of 1,25(OH)_2_D_3_ at 1 week to 4 months and whole PTH at 1 week and 1 month became significantly higher in the denosumab alone group than in BP pre-treated group (Fig. 1b and c).

## Discussion

In the present study, denosumab administration in the denosumab alone group caused: 1) strong inhibitory effects on bone resorption from as early as 1 week, 2) a slight decrease in Ca at 1 week and 1 month and a significant increase in 1,25(OH)_2_D_3_ and PTH at 1 week, followed by a gradual decrease, and 3) mild inhibitory effects on bone formation markers during the observation period. On the other hand, in the BP pre-treated group, denosumab administration resulted in: 1) further significant inhibition of urinary NTX and serum TRACP-5b, 2) a slight decrease in BAP and P1NP, 3) a slight increase in Ca at 1 week and 1 month, and 4) no marked changes in 1,25(OH)_2_D_3_ or PTH.

We noted that the bone resorption markers serum TRACP-5b and urinary NTX were significantly lower in the BP pre-treatment group at study onset, which indicated that prior therapy with BP had effectively modulated bone resorption. However, these markers decreased significantly from as early as 1 week and leveled off at 1 week for urinary NTX and 1 month for TRACP-5b. Hence, denosumab has strong inhibitory effects on bone resorption at an early stage after therapy commencement, regardless of BP pre-treatment.

With respect to bone formation markers, both BAP and P1NP also showed lower, albeit insignificant, values in the BP pre-treated group. In the denosumab alone group, BAP decreased steadily and P1NP decreased slowly after an initial marked drop. At 4 months, both values had decreased to comparable levels in both groups. However, P1NP at 4 months remained significantly higher in the denosmub alone group. In the BP pre-treated group, the values of serum BP and P1NP decreased slightly.

Generally, bone resorption and bone formation change in parallel due to the phenomenon of coupling [13]. However, we observed that this was not the case for denosumab; regardless of previous BP treatment, bone resorption was strongly inhibited in the early stages of drug administration. In the BP pre-treated group, the inhibitory effects on bone formation markers by denosumab were not obviously evident.

Ca status is strictly regulated by intestinal Ca absorption, bone resorption, and renal re-absorption. 1,25(OH)_2_D_3_ increases intestinal Ca absorption and resorption of Ca from bone, and therefore plays a prominent role in Ca regulation along with PTH [13, 14]. PTH also increases bone resorption and the production of 1,25(OH)_2_D_3_ [14]. Ca absorption occurs through changes in the level of 1,25(OH)_2_D_3_ that must be synthesized *de novo* in response to PTH [14]. As the half life of 1,25(OH)_2_D_3_ is comparatively short, the regulation of Ca, PTH, and 1,25(OH)_2_D_3_ levels is usually strictly regulated in the body.

Shiraki et al. have reported that serum 1,25(OH)_2_D_3_ and PTH levels transiently increased after alendonate administration by a yet unknown mechanism [15]. We speculated that the reasons for the changes in 1,25(OH)_2_D_3_ and PTH caused by BP therapy were decreased Ca. Furthermore, increased 1-25(OH)_2_D_3_ caused: 1) PTH receptor increase [16], 2) accelerated PTH action, 3) further increase in Ca, and 4) subsequent decreased PTH and 1,25(OH)_2_D_3_ levels [15]. In our study, in the denosumab alone group, 1,25(OH)_2_D_3_ and PTH also significantly increased after denosumab treatment. It is conceivable that a similar mechanism is involved by which denosumab strongly inhibits bone resorption, resulting in immediate and significant 1,25(OH)_2_D_3_ and PTH increases.

The most important finding in this study was that in the BP pre-treated group, regardless of further inhibiton of bone resorptive markers by denosumab therapy, 1,25(OH)_2_D_3_ did not increase and PTH tended to decrease. However, the mechanisms for such phenomena remain unknown.

The limitations of this study are 1) a small sample size, 2) short follow-up period, and 3) only a tendency of serum Ca changes may have been demonstrated due to the small cohort.

## Conclusion

In conclusion, denosumab has a strong inhibitory effect on bone resorption markers, although its inhibitory effects on bone formation markers are weak. Levels of 1,25(OH)_2_D_3_ and PTH were temporarily increased by denosumab treatment in the denosumab alone group. On the other hand, the values of these parameters did not change further as bone absorptive markers became significantly inhibited in the BP pre-treated group.
